# Analysis and validation of a Parkinson’s disease register as a recruitment tool for clinical studies

**DOI:** 10.1186/1745-6215-12-S1-A118

**Published:** 2011-12-13

**Authors:** Camille B Carroll, Amy Palmer, Christine Cosby, John P Zajicek

**Affiliations:** 1 Peninsula College of Medicine and Dentistry, Dept Clinical Neurobiology, University of Plymouth, John Bull Building, Tamar Science Park, Plymouth, Devon, PL6 8BU, UK; 2 South West Dementias and Neurodegenerative Diseases Research Network, ITTC Building, Tamar Science Park, Plymouth, Devon, PL6 8BX, UK; 3 Peninsula College of Medicine and Dentistry, University of Plymouth, Dept Clinical Neuroscience, ITTC Building, Tamar Science Park, Plymouth, Devon, PL6 8BX, UK

## Background

Many patients with Parkinson’s disease (PD) are not afforded the opportunity to participate in clinical studies. A register of research-interested patients could improve involvement. We have established a register of research-interested PD patients within the South West of England, with pragmatic inclusion criteria and multiple routes of recruitment.

## Purpose

To determine whether a register of PD patients interested in research could be established in a resource-efficient manner, and whether in comparison with traditional recruitment methods, the register would provide a more representative patient cohort and facilitate rapid and inclusive recruitment to clinical studies.

## Methods

We undertook a comprehensive analysis of the register three years after it was initiated, including documentation of the pitfalls and benefits of its establishment, investigation of its utility as a recruitment tool and a survey of recruiters.

## Results

There were 529 active participants (M:F = 1.6:1) (589 recruits, 60 withdrawn): mean age 71.4 yrs; mean disease duration 8.8 yrs from symptom onset, 7.2 yrs from diagnosis. 30% of register participants were self-referred; 70% were recruited by a healthcare practitioner. Local factors such as the availability of research support staff influenced recruitment. Response rate to annual questionnaires was 86.5%. There was a self-reported PD diagnosis rate of 92% at baseline, 88% at month 12 and 83% at month 24. Total staff time required for pack preparation, recruitment and data entry was 15 minutes for each new recruit, and 5 minutes for each follow-up questionnaire. 85% of recruiters felt the register was a useful means of facilitating research and providing data for planning of service provision. In our feasibility study, a single mailing to participants resulted in a final recruitment rate that was double that achieved by traditional face-to-face recruitment.

## Limitations

Despite 30% of participants self-referring to the register, all patients on the register were being seen in secondary care, either by a neurologist (40%) or a geriatrician with a specialist interest in movement disorders (60%). We have therefore yet to demonstrate access to a population that could not be accessed by traditional secondary-care based recruitment methods, and will be targeting register recruitment specifically in primary care to address this.

## Conclusions

We have established a register of research-interested PD patients in a resource-efficient and pragmatic manner, which has the potential to maximise inclusivity and clinical research opportunities.

